# Thalassemia and Venous Thromboembolism

**DOI:** 10.4084/MJHID.2011.025

**Published:** 2011-05-25

**Authors:** Julien Succar, Khaled M. Musallam, Ali T. Taher

**Affiliations:** Department of Internal Medicine, Hematology-Oncology Division, American University of Beirut Medical Centre, Beirut, Lebanon

## Abstract

Although the life expectancy of thalassemia patients has markedly improved over the last few decades, patients still suffer from many complications of this congenital disease. The presence of a high incidence of thromboembolic events, mainly in thalassemia intermedia, has led to the identification of a hypercoagulable state in these patients. In this review, the molecular and cellular mechanisms leading to hypercoagulability in thalassemia are highlighted, with a special focus on thalassemia intermedia being the group with the highest incidence of thrombotic events as compared to other types of thalassemia. Clinical experience and available clues on optimal management are also discussed.

## Introduction:

The thalassemias, a group of inherited disorders of hemoglobin synthesis, are the most common monogenetic disease worldwide.^1^ Extremely diverse phenotypes exist within the thalassemia syndromes. At one end of the spectrum is thalassemia minor, a clinically silent, mildly hypochromic and microcytic anemia. At the other end is thalassemia major (TM) which refers to those patients whose clinical course is characterized by profound anemia, who are presented to medical attention in the first year of life, and who subsequently require regular blood transfusions for survival.^2^ The term thalassemia intermedia (TI) was first suggested to describe patients who had clinical manifestations that were too severe to be termed minor yet too mild to be termed major, although there remains substantial overlap between the three conditions.^3^ Our understanding of the molecular and pathophysiological mechanisms underlying the disease process in patients with TI has substantially increased over the past decade.^4^ Three main factors highlight the pathophysiology of TI, ineffective erythropoiesis, chronic anemia/hemolysis, and iron overload secondary to increased intestinal absorption.^4^ However, the extreme diversity in phenotypic expression in TI patients led to a wide variation in observed clinical complications.^5^ Among the clinical complications of TI that were found to occur at a higher rate than in patients with TM are thromboembolic events (TEE).^6^–^7^ We herein review hypercoagulability in patients with thalassemia and its translation into clinical TEE, with special emphasis on TI patients.

## Pathophysiology:

Hypercoagulability in patients with thalassemia has been attributed to several risk factors.^8^ It is often a combination of these factors that leads to TEE.

It is widely accepted that patients with thalassemia have chronically activated platelets, and enhanced platelet aggregation,^9^ as confirmed by the increased expression of CD62P (P-selectin) and CD63, markers of in vivo platelet activation.^10^–^11^ Platelets in thalassemia have a shorter life span, particularly in splenectomized patients, due to enhanced consumption.^12^ It has also been shown that splenectomized TM and non-splenectomized TI patients have 4 to 10 times more metabolites of prostacyclin (PGI2) and thromboxane A2 (TXA2), both markers of hemostatic activity, than controls. However, no significant difference was found between TM and TI patients.^13^ Thus, the higher rate of TEE in patients with TI compared to TM cannot be explained by abnormalities in platelet function, and shifts the attention to the pathogenic role of red blood cells (RBCs) described hereafter. Splenectomy also leads to higher platelet count.^14^–^15^

Furthermore, the oxidation of globin subunits in thalassemia erythroid cells leads to the formation of hemichromes,^2^ which precipitate, instigating heme disintegration and the eventual release of toxic nontransferrin-bound iron species.^16^ The free iron in turn catalyzes the formation of reactive oxygen species, leading to oxidation of membrane proteins and formation of red-cell “senescence” antigens like phosphatidylserine,^17^ which cause the thalassemic RBCs to become rigid, deformed, and to aggregate, resulting in premature cell removal.^18^ Thalassemic RBCs with negatively charged phospholipids have been shown to increase thrombin generation,^19^–^20^ as evidenced by studies using the protein annexin V, a protein with high affinity and specificity for anionic phospholipids.^20^ Studies have also shown that splenectomized patients have a higher number of negatively charged RBCs and increased thrombin generation.^21^–^22^ Thalassemic RBCs also have enhanced cohesiveness and aggregability. These abnormalities have been reduced to normal range after introducing a blood transfusion.^23^

TI patients were also found to have higher levels of procoagulant microparticles of RBC, leukocytic, and endothelial origins compared to controls.^24^

The presence of other peripheral blood elements in thalassemics such as E-selectin (ELAM-1), intercellular adhesion molecule-1 (ICAM-1), von Willebrand factor (VWF) and vascular cell adhesion molecule-1 (VCAM-1) indicates that endothelial injury or activation may be an aspect of the disease, aiding in the recruitment of white blood cells and RBCS, promoting thrombosis.^25^–^26^ Studies have demonstrated that RBCs from TM and TI patients show increased adhesion to cultured endothelial cells (EC).^27^ Butthep et al. showed that in addition to the presence of EC expressing adhesion molecules and tissue factor in the circulation, thalassemia patients also have decreased levels of Protein C and Protein S compared with normal.^28^ Prothrombin fragment 1.2 (F1.2), a marker of thrombin generation, is elevated in patients with TI.^29^

Prothrombotic mutations do not play a role in the hypercoagulability of thalassemia. Studies in Italy and Lebanon have revealed that the presence of factor V Leiden, prothrombin, and methylene tetrahydrofolate reductase mutations did not contribute to the risk of TEE in patients with thalassemia.^30^–^31^ The presence of cardiac, hepatic, or endocrine dysfunction may also contribute to the hypercoagulability in thalassemia.^8^

## Clinical Experience:

**Figure 1.**
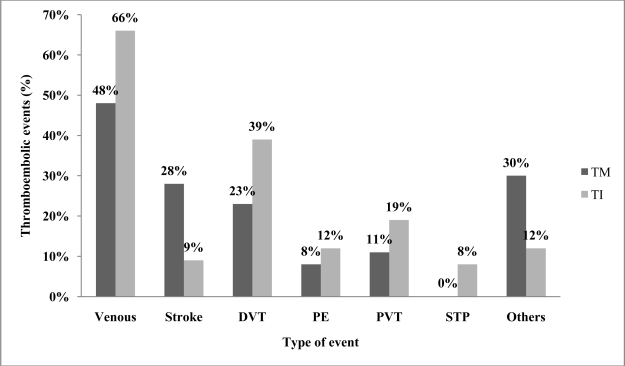
Type of thromboembolic events in thalassemia intermedia (TI) vs. thalassemia major (TM).^7^ DVT = deep vein thrombosis, PE = pulmonary embolism, PVT = portal vein thrombosis, STP = superficial thrombophlebitis.

Epidemiological data on TEE in thalassemia are scarce. Borgna-Pignatti et al. surveyed nine Italian pediatric thalassemia centers, observing that 4% of the 683 patients with TM and 9.6% of the 52 patients with TI had experienced a TEE.^6^ The same group showed six years later that 1.1% of 720 patients with TM in seven Italian centers had thrombosis^32^. Cappellini et al. followed-up 83 patients with TI over 10 years, 82 of whom were splenectomized, and found that 29% (24/83) experienced a venous TEE.^22^ One study directly implicated TEE as the cause of death in 2.5% of transfusion-dependent thalassemia patients.^33^ After examining data from 8,860 patients in the Mediterranean area and Iran, Taher et al. observed that TEE occurred 4.38 times (95%CI 3.14 – 6.10, *P* < 0.001) more frequently in TI than TM, with more venous events occurring in TI and more arterial events occurring in TM (Figure 1).^7^ It was found that 14% of mortalities in the whole group were due to TEE^7^. Age above 20 years, splenectomy, family history of TEE, and previous TEE were identified as the main risk factors for thrombosis in TI. Furthermore, the study showed that 68% of TI patients that had a TEE had an average hemoglobin level of < 9 g/dl and only 33% were receiving regular blood transfusions, whereas 94% were splenectomized.^7^ Moreover, patients receiving aspirin therapy had a significantly lower rate of recurrent TEE.^7^

The evidence for brain involvement in thalassemia dates back to 1972 where 20% of 138 TM patients in Greece were found to have neurological deficits compatible with transient ischemic attacks (TIAs).^34^ Further evidence of TIAs causing neurological symptoms, such as headaches, hemiparesis, and seizures was shown in 2.2% of patients with TM in Italy.^6^ Although overt stroke occurs more frequently in TM than TI (28% vs. 9%, respectively),^7^ it has been shown that as many as 37.5% of patients with TI have asymptomatic brain damage on magnetic resonance imaging (MRI).^34^ A more recent study determined that splenectomized adults with TI show a rate of silent white matter lesions as high as 60%.^35^ The occurrence and multiplicity of the lesions were associated with older age (mean age of 36.1 years for lesion positive-patients vs. 26.1 years for lesion-negative patients) and transfusion naivety (83.3% of lesion-positive patients have never had a transfusion vs. 25% of lesion-negative patients).^35^

In order to obtain much needed clinical data on the optimal management of patients with TI, the Overview on Practices in Thalassemia Intermedia Management Aiming for Lowering Complication rates Across a Region of Endemicity (OPTIMAL CARE) study evaluated 584 patients with TI at six comprehensive care centers (Lebanon, Italy, Iran, Egypt, United Arab Emirates, and Oman) for the associations between patient and disease characteristics, treatment received, and the rate of complications.^5^ The study analyzed complications against the parameters of age, gender, serum ferritin level, hemoglobin level, splenectomy, transfusion, hydroxyurea, and iron chelation therapy. Thrombosis was the 5^th^ most common complication, affecting 14% of the patient population. On multivariate analysis, splenectomy, age above 35 years, and a serum ferritin level ≥ 1000 μg/l were associated with a higher risk for thrombosis.^5^ Conversely, a positive history of transfusion and a hemoglobin level ≥ 9 g/dl were found to be protective against thrombosis (Table 1).^5^ Another study further confirmed the higher occurrence of thrombosis with advancing age.^36^

In an effort to further understand the effect of splenectomy on TEE, a sub-study of the OPTIMAL CARE examined the characteristics of splenectomized patients with TI who develop TEE aiming to identify high-risk patients who deserve further consideration for preventive strategies.^37^ Splenectomized patients with documented TEE (Group I, n = 73) were age- and sex-matched with splenectomized patients without TEE (Group II) and non-splenectomized patients without TEE (Group III). The study determined that splenectomized TI patients who experience TEE are characterized by high nucleated RBC (≥ 300 x 10^6^/l) and platelet counts (≥ 500 x 10^9^/l), are more likely to have evidence of pulmonary hypertension (PHT) and be transfusion naïve. As such, the authors suggest that splenectomized TI patients at risk of developing TEE may be identified early on by these laboratory markers, presence of PHT, and transfusion status.^37^ The study further examined how long it took for a TEE to develop following splenectomy and found the median time to thrombosis to be 8 years.^37^ The delay indicates that the etiology behind TEE in splenectomized patients with TI is not an acute complication, but a manifestation of a chronic underlying process, further emphasizing the need for a long-term treatment modality for prevention.^37^

## Management:

The reduction of procoagulant RBCs by transfusion has been suggested as a reason behind the lower rate of TEE in transfused vs. non-transfused patients.^5^,^7^,^35^,^37^ As such, transfusion therapy may be worthwhile to prevent the occurrence of TEE, especially in TI patients in whom current practice does not necessarily recommend transfusions. However, this needs to be prospectively evaluated.

Since splenectomy is a major contributor to TEE in patients with thalassemia, reassessment of the procedure and appropriate risk benefit-evaluation is called for. This is also important because of its correlation with other complications such as osteoporosis, PHT, cholelithiasis, hypothyroidism, diabetes mellitus, heart failure, increased susceptibility to infection, and leg ulcers in TI.^5^

The literature lacks proper evidence on the role of antiplatelet or anticoagulant agents in the management of thalassemia.^8^ The lower recurrence of TEE in TI patients who took aspirin after their first TEE, when compared to those who did not, suggests a potential role for aspirin.^7^ Moreover, the association of higher platelet counts with TEE in patients with TI further suggests a role for aspirin in this patient population.^37^

Fetal hemoglobin inducing agents like decitabine and hydroxycarbamide were also shown to lower plasma markers of thrombin generation.^29^ Hydroxycarbamide may modulate hypercoagulability in several ways, it may reduce phospholipid expression on the surface of RBCs and platelets, and decrease RBC adhesion to thrombospondin, a thrombin sensitive protein.^29^ It may also decrease leukocyte count, particularly monocytes expressing transcription factor, in addition to being a nitric oxide donor.^38^

It is recommended that each patient be assessed individually and assigned a personalized thrombotic risk based on intrinsic (thalassemia type, number of circulating RBCs, etc) and extrinsic factors (splenectomy, transfusion status, etc).^29^ High nucleated RBC and platelet counts, evidence of PHT, and transfusion naivety can be used as indicators of TEE for splenectomized patients with TI and could be practical in the clinical setting.^37^ Such a risk-assessment model (RAM) would be valuable in identifying high-risk patients and targeting them for further testing. The RAM could serve as a guideline for preventative treatment and significantly decrease morbidity and mortality.^29^ Other diagnostic tests are being explored to help identify patients at risk, with promising preliminary results.^39^

## Conclusions:

The hypercoagulable state in thalassemia is due to multiple elements, a combination of which is often the drive behind a clinical TEE. Splenectomy and transfusion naivety are increasingly highlighted as important risk factors for TEE, especially in patients with TI. An individualized approach is recommended to establish an optimal strategy for preventing the occurrence of this complication if thalassemia.

## Figures and Tables

**Table 1.** Predictors of thrombosis in the OPTIMAL CARE study.^5^

**Parameter**	**Adjusted OR**	**95% CI**	***P-value***
Age > 35	2.59	1.39 – 4.87	0.003
Female	1.27	0.74 – 2.19	0.387
Hemoglobin ≥ 9 g/dl	0.41	0.23 – 0.71	0.001
Ferritin ≥ 1000 μg/L	1.86	1.09 – 3.16	0.023
Splenectomy	6.59	3.09 – 14.05	< 0.001
Transfusion	0.28	0.16 – 0.48	< 0.001
Hydroxyurea	0.56	0.28 – 1.10	0.090
Iron chelation	0.97	0.56 – 1.68	0.912
